# Supplementary figures and images for: Analysis of KIR gene variants in The Cancer Genome Atlas and UK Biobank using KIRCLE
Source: BMC Biol. 2022 Aug 24;20:191. doi: 10.1186/s12915-022-01392-2 (PMC9400285; doi:10.1186/s12915-022-01392-2)

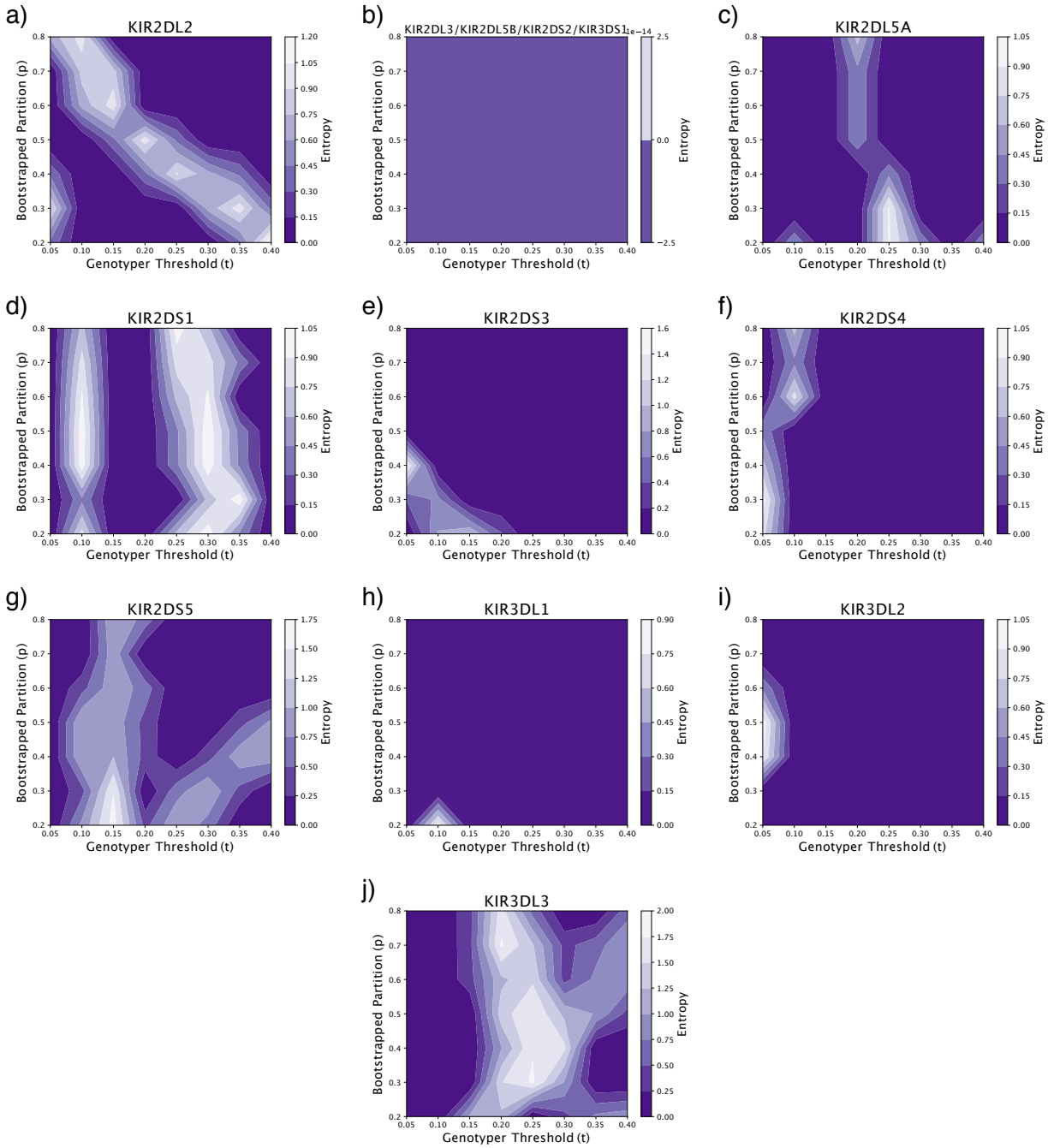

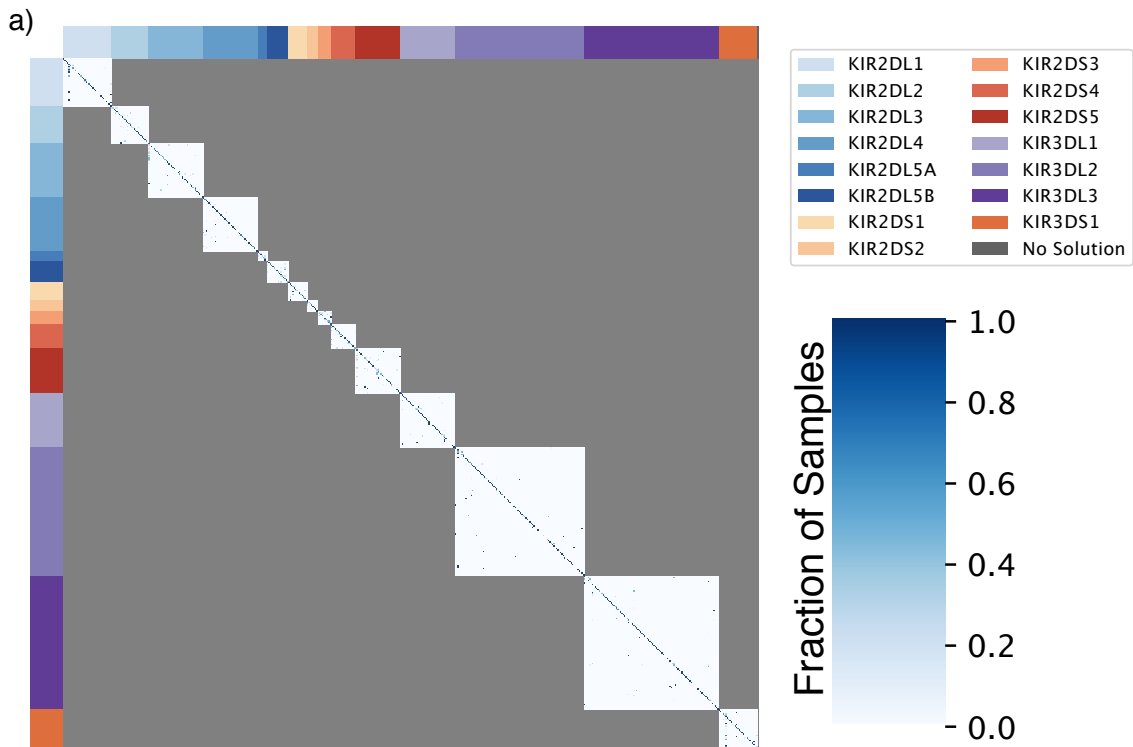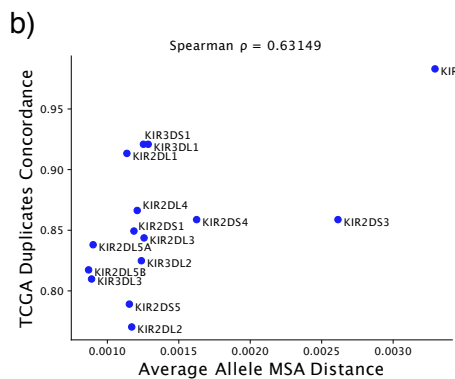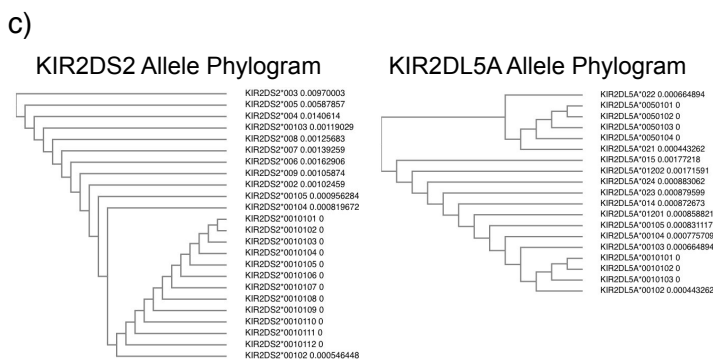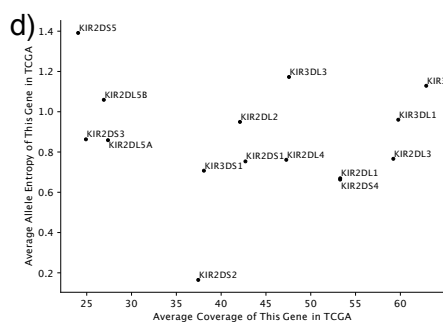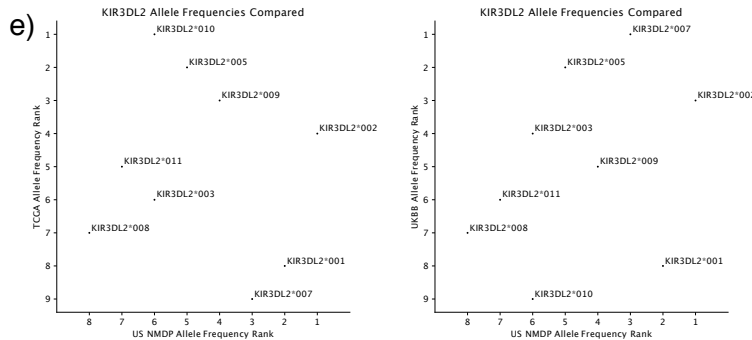

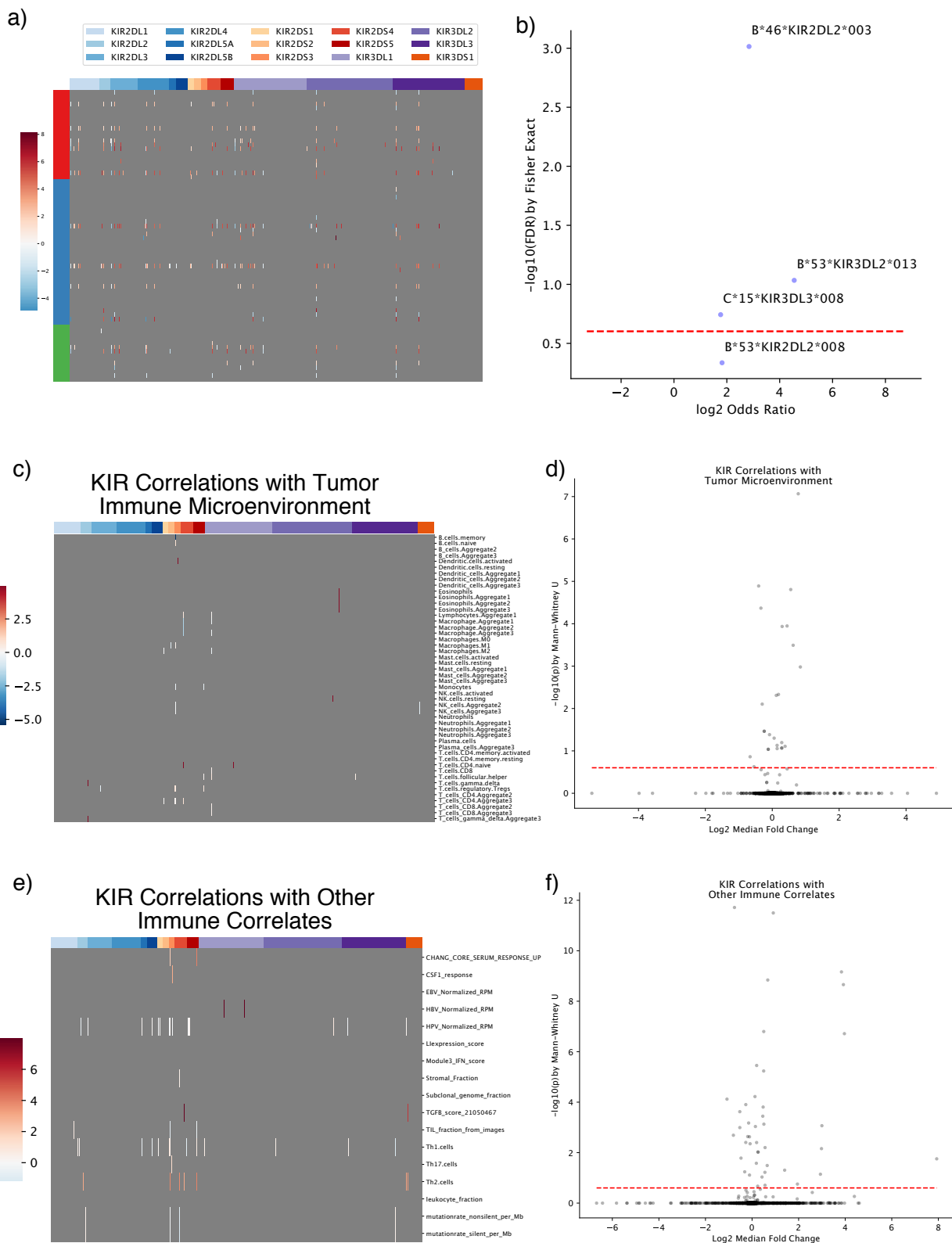

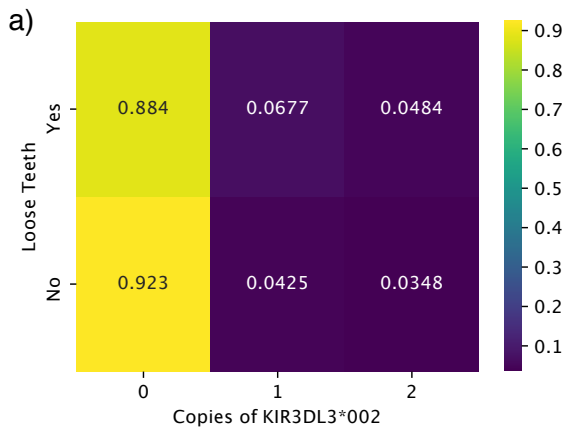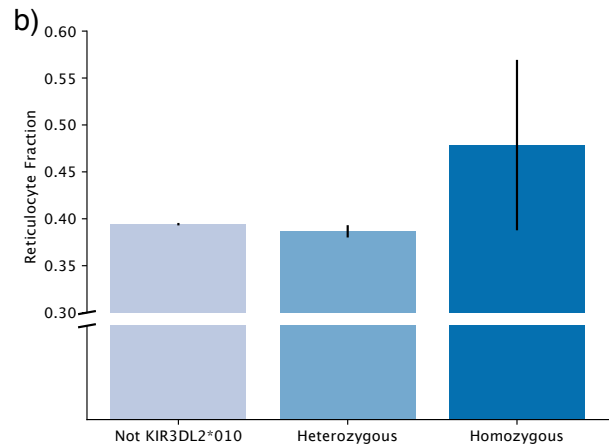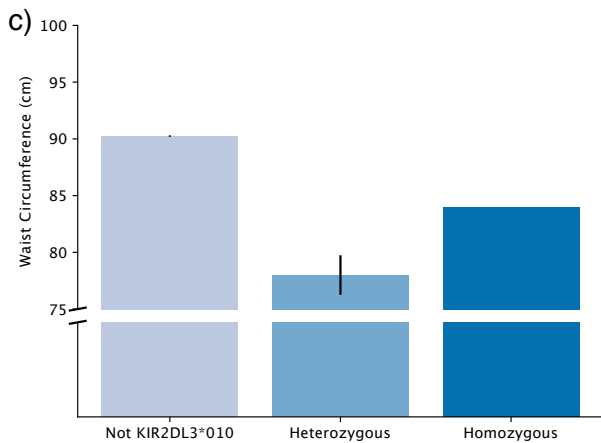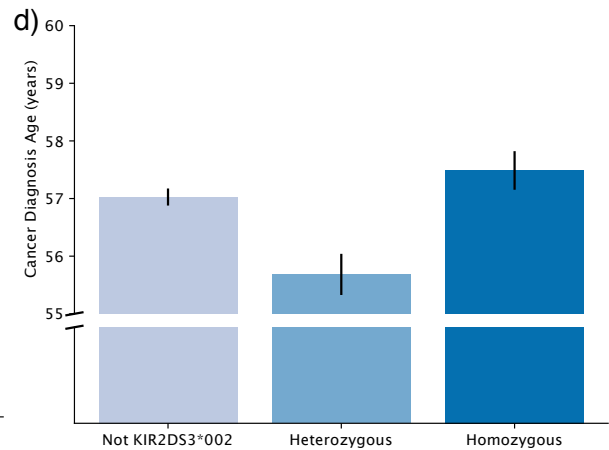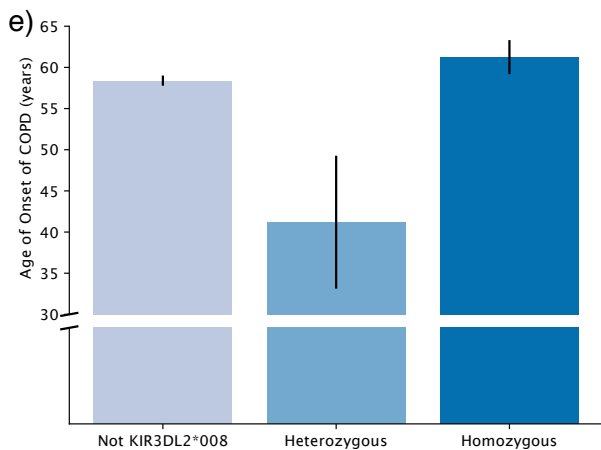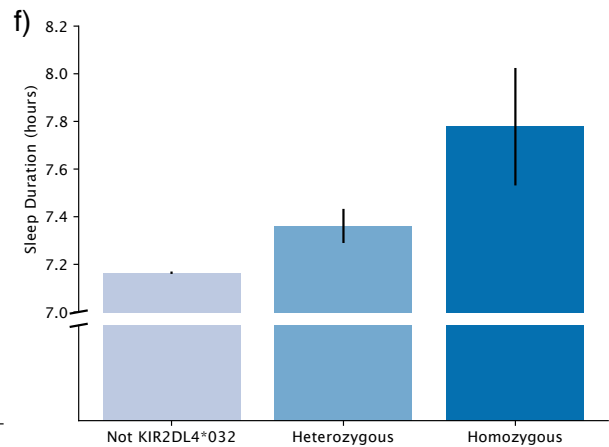

Supplement: Supplementary file 2 — Additional file 2: Supplementary Figure S1. Validation of KIRCLE’s Consistency. Contour plots demonstrating the effect of varying p and t on consistency of genotype calling, as quantified by entropy, for (a) KIR2DL2; (b) KIR2DL3, KIR2DL5B, KIR2DS2, & KIR3DS1; (c) KIR2DL5A; (d) KIR2DS1; (e) KIR2DS3; (f) KIR2DS4; (g) KIR2DS5; (h) KIR3DL1; (i) KIR3DL2; (j) and KIR3DL3. Supplementary Figure S2. Validation of KIRCLE’s Accuracy. (a) Confusion matrix depicting KIRCLE’s consistency in KIR genotype inference between 531 samples and their biological replicates in TCGA. (b) Scatterplot demonstrating that the concordance of KIR genotypes between TCGA replicates in each KIR gene positively correlates with sequence “dissimilarity” (as measured by average MSA distance) between alleles of that KIR gene. (c) Example MSA phylograms for KIR2DS2 (left) and KIR2DL5A (right) demonstrate different degrees of sequence similarity between different alleles of the same KIR gene. (d) Average entropy versus average depth of coverage in TCGA for each KIR gene. (e) Comparison of allele frequency ranks among the 9 KIR3DL2 alleles observed in the US NMDP with their frequency ranks in TCGA (left) and UK Biobank (right). Supplementary Figure S3. KIR Correlations with Molecular Markers. (a) Heatmap of the log2-odds-ratios of KIR allele correlations with HLA alleles in UK Biobank. Correlations with Fisher’s Exact FDR > 0.25 were masked. (b) Volcano plot of KIR allele correlations with HLA alleles in TCGA. (c) Heatmap of log2-median-fold-changes in tumor immune infiltrate composition estimates stratified by KIR alleles in TCGA. Mann-Whitney-U FDR > 0.25 correlations were masked. (d) Volcano plot of KIR allele correlations with differences in tumor immune infiltrate composition in TCGA. (e) Heatmap of log2-median-fold-changes in other immune-related molecular signatures and markers stratified by KIR alleles in TCGA. Mann-Whitney-U FDR > 0.25 correlations were masked. (f) Volcano plot of KIR allele correl [file 12915_2022_1392_MOESM2_ESM.pdf]
